# *Camellia debaoensis* (Theaceae), a new species of yellow camellia from limestone karsts in southwestern China

**DOI:** 10.3897/phytokeys.135.38756

**Published:** 2019-11-28

**Authors:** Renchuan Hu, Sujuan Wei, Yongqing Liufu, Yunkai Nong, Wei Fang

**Affiliations:** 1 Guangxi Institute of Chinese Medicine and Pharmaceutical Science, Nanning, Guangxi 530022, China Guangxi Institute of Chinese Medicine and Pharmaceutical Science Nanning China; 2 Guangxi Institute of Botany, Guangxi Zhuang Autonomous Region and the Chinese Academy of Sciences, Guilin, Guangxi 541006, China Guangxi Institute of Botany, Chinese Academy of Sciences Guilin China; 3 Guangxi Museum of Natural History, Nanning, Guangxi 530012, China Guangxi Museum of Natural History Nanning China; 4 Kunming Institute of Botany, Chinese Academy of Sciences, Kunming, Yunnan 650201, China Kunming Institute of Botany, Chinese Academy of Sciences Kunming China

**Keywords:** *
Camellia
*, China, limestone flora, taxonomy, Theaceae

## Abstract

*Camellia debaoensis* R.C.Hu & Y.Q.Liufu, **sp. nov.** is described and illustrated as a new species from southwestern Guangxi, China. It is morphologically similar to *Camellia pubipetala* Y. Wan & S. Z. Huang, *C. mingii* S.X. Yang and *C. tuyenquangensis* D.V. Luong, N.N.H. Le & N. Tran, but it differs from these species in having glabrous young branches, glabrous petiole, glabrous sepals, glabrous petals, glabrous stamens and glabrous ovary, 10 petals, cylindrical ovary and style 3-lobed to 1/6 style length.

## Introduction

Guangxi Zhuang Autonomous Region of southern China is an area noted for its karst landscapes (Hou et al. 2010). The limestone region in southwestern Guangxi harbors very high levels of biological diversity and is recognized as one of 20 centers of plant endemism in China (Myers et al. 2000, López-Pujol et al. 2011). Yellow camellia, a subgroup of *Camellia* (Theaceae), are characterized by yellow, waxy and shiny petals (Chang and Ren 1998). Because of their beautiful flowers and useful chemical constituents, yellow camellias have considerable economic value in breeding, as well as traditional Chinese medicine and commercial tea production (He et al. 2016). Before 2007, fewer than 20 yellow camellias from China were recognized, according to the literatures (Ye and Xu 1992; Chang and Ren 1998; Min 2000; Min and Bruce 2007). Most of them are only distributed in southwestern Guangxi, which had been considered as a center of diversity of the yellow camellia. In recent years, many new species of yellow camellia have been reported from northern Vietnam and southern China (e.g. Tran and Nguyet 2005; Orel 2006; Orel and Wilson 2010, 2012; Orel et al. 2012, 2013, 2014a, b; Tran et al. 2012; Tran and Luong 2013; Huang et al. 2014; Orel and Curry 2015; Tran and Le 2015; Luu et al. 2015; Dung et al. 2016; Luong and Le 2016; Luong et al. 2016a, b; Le et al. 2017; Nguyen et al. 2018; Liu et al. 2019), increasing the total to more than 50 species (Tran et al. 2019) and making northern Vietnam another center of yellow camellia diversity. Generally, yellow camellias are rare and highly endemic due to their small population, narrow distribution and excessive gathering. Recently, almost all Chinese yellow camellia species were categorized as Critically Endangered, Endangered, or Vulnerable species in the Threatened Species List of China’s Higher Plants (Qin et al. 2017).

During our floristic survey in limestone karsts of Debao County, southwestern Guangxi, in 2015, we collected several specimens from a population of *Camellia* with yellow flowers. In the following three years, this population was documented for flowering and fruiting regularly at the same locality. Morphological comparison between the newly collected specimens and other yellow camellias suggested that the specimens from Debao differed from all the previously described species. Therefore, we here describe this material as a new species.

## Materials and methods

Several specimens were collected at the entrance of one of the karst caves of Debao County, Jingde Town, Tuoliang village from 2015 to 2018, and were deposited in the herbaria GXMI, IBK, NHMG, KUN. The morpho-photographs of the plants were taken with a Panasonic LX100 camera. This material was confirmed as a new species based on detailed comparison with all other heretofore known yellow camellias, including specimens deposited at PE, KUN, IBSC, IBK, GXMI, HIB, SYS, GXMG, and description from botanical websites (e.g. http://www.cvh.ac.cn/, https://plants.jstor.org/). Herbarium acronyms follow Thiers (2018). The morphological characters were measured using M & G ARL96004.

## Taxonomic treatment

### Camellia debaoensis

Taxon classificationPlantaeEricalesTheaceae

R.C.Hu & Y.Q.Liufu sp. nov.

1B2A2CD9-6EC8-5FE0-B38D-A5503A72D38F

urn:lsid:ipni.org:names:77203187-1

Figs 1 , 2

#### Diagnosis.

Morphologically, the new species is similar to *Camellia pubipetala* Y. Wan & S.Z. Huang, *C. mingii* S.X. Yang and *C. tuyenquangensis* D.V. Luong, N.N.H. Le & N. Tran, but it differs from these species in having glabrous young branches, glabrous petiole, glabrous sepals, glabrous petals, glabrous stamens and glabrous ovary, 10 petals, cylindrical ovary and 3-lobed to 1/6 style length.

#### Type.

**China.** Guangxi Zhuang Autonomous Region: Debao County, Jingde Town, Tuoliang village, at the entrance of one of karst caves, rare, 23°29'23.12"N, 106°9'47.27"E, 760 m a.s.l., 13 January 2017 (fl.), *R.C. Hu HRC170113002* (holotype: GXMI!, isotypes: GXMI!, KUN!, NHMG! and IBK!).

**Figure 1.**
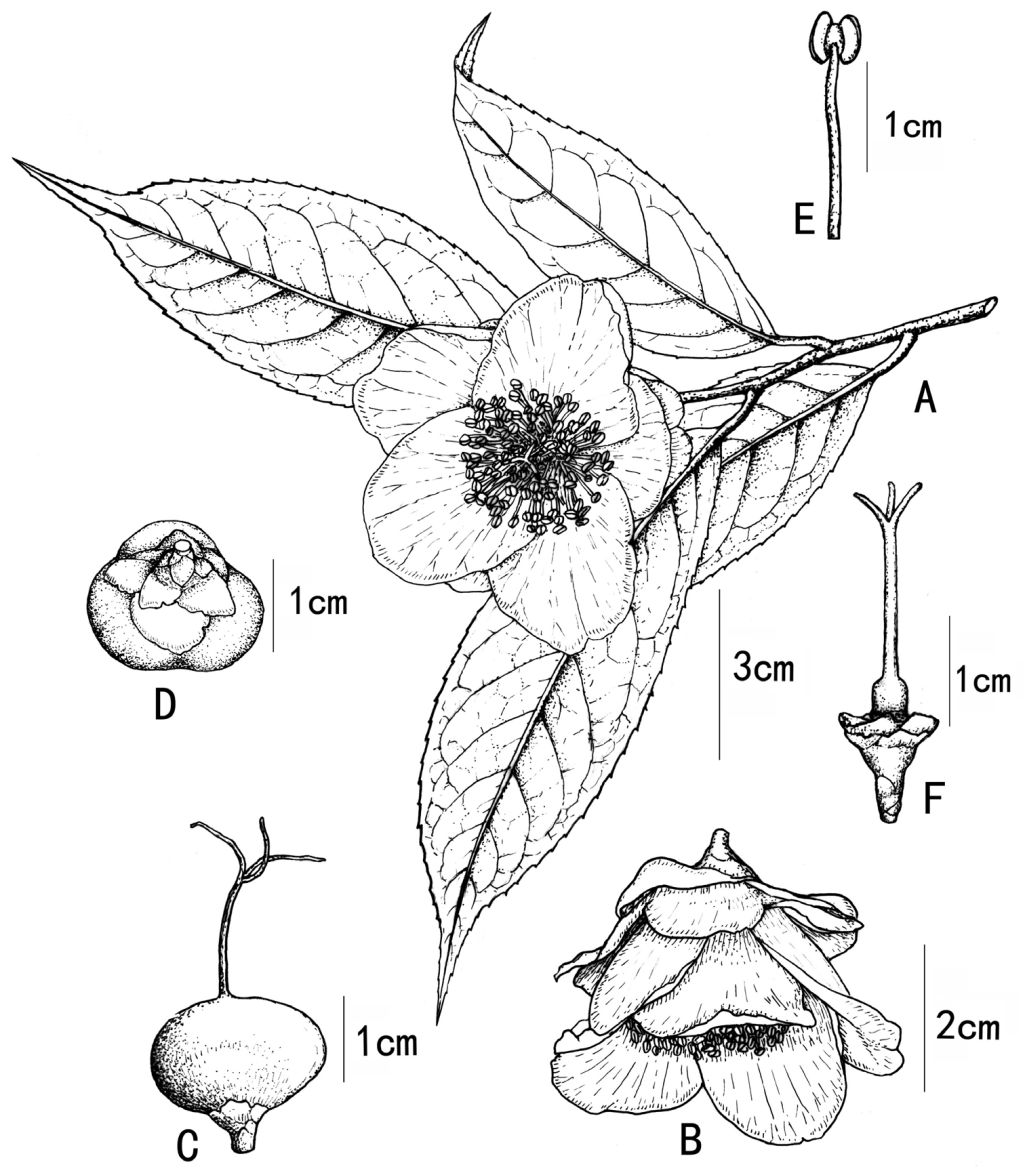
*Camellia debaoensis* R.C.Hu & Y.Q.Liufu, sp. nov. **A** flowering branch **B** lateral view of flower **C** fruit and style **D** fruit, sepals and bracteoles **E** stamen **F** pistil. Drawn by Xincheng Qu.

**Figure 2.**
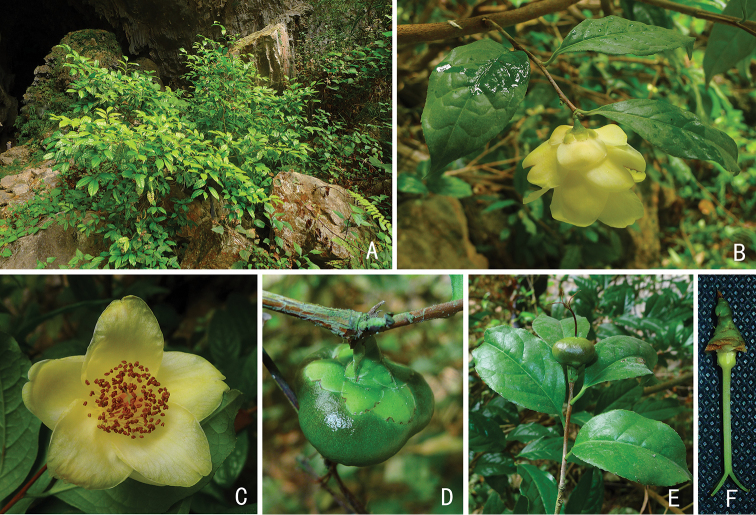
*Camellia debaoensis* R.C.Hu & Y.Q.Liufu, sp. nov. **A** habit **B** flowering branch **C** face view of flower **D** fruit, sepals and bracteoles **E** fruiting branch **F** pistil. Photographed by Renchuan Hu.

#### Description.

Shrubs, 1–3 m tall. Young branches cylindrical, thick, glabrous, yellowish brown or grayish brown, and current year branchlets purplish red. Leaf blade leathery, ovate to long ovate, 6–13 × 3–5 cm, adaxial surface dark green and glabrous, abaxial surface pale green, brown glandular punctuate and veins sparsely spreading villous, veins abaxially elevated and adaxially impressed, secondary veins 5–6 on each side of midvein and connected at the proximal edge, base cuneate to broadly cuneate, apex caudate tip, margin serrulate; petiole 5–12 mm long, glabrous. Flowers subterminal axillary, solitary, 3–4.5 cm diam. Pedicel ca. 4(–6) mm long, thick; bracteoles 4 (or 5), unequal, 1–3 × 2–4 mm, appressed and covering pedicel, oval-triangle, leathery, green and glabrous, margin ciliolate. Sepals 5 (–6), semiorbicular to broadly ovate, 3–5 × 5–8 mm, leathery, glabrous, lightly yellow and occasionally with pink patches, fruiting stage green, margin ciliolate. Petals 10, in three whorls of 3–4 petals, golden yellow, glabrous; outer 3 or 4 petals suborbicular, occasionally with pink patches, 0.7–1.1 × 1 cm; inner orbicular-ovate or oval, 1.2–1.8 × 1.2–2.6 cm, basally connate for 1–3 mm. Stamens numerous, glabrous, ca. 2 cm long; anthers ca. 3 × 1mm; outer filaments connate ca. basal 1/4, ca. 1.6 cm, inner filaments nearly distinct, ca. 1.7 cm. Ovary cylindrical, ca. 2 mm in diam., glabrous, 3-loculed; style 2 cm long, glabrous, base connate, apex 3-lobed to 1/6 style length. Capsule triangle oblate, glabrous, 1.4–1.6 × 1.6–2.8 cm; Seeds brown, hemispherical, pubescent.

#### Phenology.

Flowering from December to February of the next year; fruiting from July to August.

#### Distribution and habitat.

**Figure 3.**
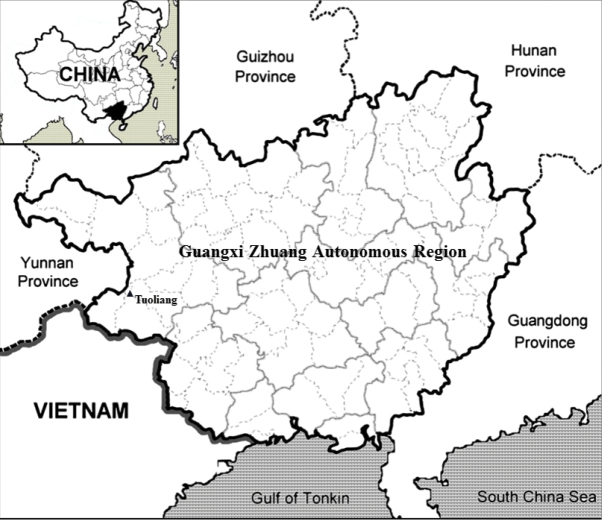
Map showing distribution of *Camellia debaoensis* R.C.Hu & Y.Q.Liufu, sp. nov. in southwestern Guangxi, China.

*Camellia debaoensis* grows at the entrance of one of the limestone caves in the karst region of Debao County (Fig. 3), Guangxi, China, accompanied by *Ageratina adenophora* (Sprengel) R. M. King & H. Robinson (Compositae), *Boehmeria penduliflora* Wedd. ex Long (Urticaceae), *Fallopia multiflora* (Thunb.) Harald (Polygonaceae), *Flueggea virosa* (Roxb. ex Willd.) Voigt (Euphorbiaceae), *Pteris vittata* L. (Pteridaceae), *Ficus tikoua* Bur (Moraceae), and Pueraria montana (Lour.) Merr. var. lobata (Willd.) Maesen et S. M. Almeida ex Sanjappa et Predeep (Fabaceae).

#### Conservation status.

According to currently available data, *Camellia debaoensis* is only found in its type locality and there are only nine adult trees and four saplings, with the distribution restricted to a very limited region (less than 200 m^2^). Considering this situation, we consider *Camellia debaoensis* as ‘Critically Endangered’ (CR) based on the IUCN categories and criteria (IUCN 2017).

#### Additional specimens examined.

China. Guangxi Zhuang Autonomous Region: Debao County, Jingde Town, Tuoliang village, at the entrance of karst cave, rare, ca. 760 m a.s.l., 13 Jan. 2017 (fl.), *R.C. Hu HRC170113001* (GXMI!); the same locality, 21 May 2016 (fr.), *R.C. Hu HRC170521001* (GXMI!); the same locality, 25 Dec. 2015 (fl.), *R.C. Hu & Y.Q. Liufu HRC151225023* (GXMI!).

#### Etymology.

The specific epithet is derived from the type locality, Debao County, Guangxi.

#### Taxonomic notes.

It is noted that there are several classification systems about taxonomic treatments of *Camellia*, represented by Ye and Xu (1992), Chang and Ren (1998), and Min and Bruce (2007). These systems have different taxonomic opinions on sectional taxonomic treatment of yellow camellias. Considering the inclusiveness of the system of Min and Bruce (2007), the new species should be placed in C. sect. Stereocarpus.

*Camellia debaoensis* resembles many other yellow-flowering camellia species with connate style, such as *Camellia pubipetala* Y. Wan & S.Z. Huang, *C. mingii* S.X. Yang, *C. tuyenquangensis* D.V. Luong, N.N.H. Le & N. Tran, *C. oconariana* Orel, Curry & Luu, *C. thuongiana* Luong, Anna Le & Lau, *C. luteocerata* Orel, *C. luteopallida* Luong, T.Q.T. Nguyen & Luu, *C. bugiamapensis* Orel, Curry, Luu & Q.D. Nguyen and *C. capitata* Orel, Curry & Luu. These species are placed in various sections within the genus including sections *Stereocarpus* Chang, *Chrysantha* Chang, *Dalatia* Orel and *Capitatae* Orel (Table 1). A key to identifying species of yellow camellia with connate style is provided below. Of these species, the new species is more similar to *C. pubipetala*, *C. mingii*, *C. tuyenquangensis* than to other species by sharing small leaflets, subterminal axillary flowers, 9–13 petals, differentiated bracts, outer filament whorl basally connate, 3-locular ovary, connate style. However, it is well distinguished from these species in having glabrous young branches, glabrous petiole, glabrous sepals, glabrous petals, glabrous stamens and a glabrous ovary (vs. villous in *C. pubipetala* and *C. mingii*), 10 petals (vs. 9–13 in *C. pubipetala*, 12–13 in *C. mingii* and 12 in *C. tuyenquangensis*), cylindrical ovary (vs. ovoid in *C. mingii* and *C. tuyenquangensis*, spherical in *C. pubipetal*) and style 3-lobed to 1/6 style length (vs. 3 (or 4)-cleft to 1/3 style length in *C. pubipetala*, 3-cleft to 1/10 style length in *C. mingii*, 3-cleft to 1/2 style length in *C. tuyenquangensis*). The comparisons between *C. debaoensis* and these species are provided in Table 2. Based on hairs, lamina length, flower size, number of carpels and other characteristics, the new species is also a taxonomic entity distinct from *C. oconariana*, *C. thuongiana*, *C. luteocerata*, *C. luteopallida*, *C. bugiamapensis* and *C. capitata* (Table 1).

**Table 1.** Morphological comparison of *C. debaoensis* with other yellow camellias with connate style. Data from Min and Bruce (2007), Orel and Wilson (2010), Orel et al. (2013, 2014a), Luong et al. (2016a, b), Le et al. (2017), Liu et al. (2019).

Taxon	Section	Leaf shape, leaf size	Pedicel length	No. of bracts	No. of petals	Style, extent to which cleft, hairiness	Ovary, carpels, hairiness	Filaments morphology
*C. capitata*	* Capitatae *	Elliptic to oval, 24.0–27.0 × 10.0–12.0 cm	Sessile	8–10	6	Apex 3-lobed, apically cleft for 1–2 mm, glabrous	3-carpellate, glabrous	Glabrous, outer filaments basally attached to inner petals for ca. 10 mm
*C. luteocerata*	* Dalatia *	Elliptic to broadly elliptic, 22.0–27.5 × 9.0–11.0 cm	Sessile	6	11–13	Apex 5-lobed, apically cleft to 1/3 style length, glabrous	5-carpellate, glabrous	Glabrous, filaments basally connate for 1/3 of their length, and partially joined to the inner petals
*C. bugiamapensis*	* Dalatia *	Elliptic, oval to widely elliptic, 18.0–27.5 × 12.0–14.0 (–15.0) cm	Sessile	7	9–11	Apex 5-lobed, apically shortly lobed, white hairs	5–6-carpellate, glabrous	Glabrous, outer filaments basally attach to inner petals for 20–25 mm
*C. luteopallida*	* Dalatia *	Elliptic to ovate, 16.0–20.0 × 5.0–9.0 cm	Sessile	6–9	12–14	Apex 3-lobed, apically cleft 3–6 mm, dense white hairs	3-carpellate, glabrous	Sparely hairy, filaments basally united to each other to form a 10–13 mm fleshy tube
*C. tuyenquangensis*	* Chrysantha *	Oblong ovate to narrow elliptic, 14.0–18.0 × 5.0–8.0 cm	10 mm	4–5	12	Apex 3-lobed, apically cleft to 1/2 style length, glabrous	3-carpellate, glabrous	Glabrous, outer filament whole basally connate for 10–14 mm, adnate to petal base
*C. thuongiana*	* Chrysantha *	Elliptic to oblong elliptic, 9.0–17.0 × 4.0–6.5 cm	8–10 mm	3–4	11–13	Apex 3-lobed, apically cleft to 1/2 style length, glabrous	3-carpellate, pubescent	Glabrous, outer filaments basally connate for 4–5 mm
*C. oconoriana*	* Chrysantha *	Narrowly elliptic, 30.0–36.5 × 8.0–8.5 cm	30–40 mm	6	6	Apex 3–5-lobed, apically cleft 9–11 mm, finely hairy proximally, glabrous distally	4–5-carpellate, densely tomentose	Glabrous, outer filaments basally connate for ca. 6 mm
*C. pubipetala*	* Stereocarpus *	Elliptic to ovate, 10.0–17.0 × 5.0–8.0 cm	Subsessile	(4–)6–8	9–13	Apex 3(or 4)-lobed, apically cleft 5–10 mm, tomentose	3(or 4)-loculed, yellowish tomentose	Distinct part pilose, outer filament whorl basally connate for ca. 1/3 of its length
*C. mingii*	* Stereocarpus *	Elliptic-ovate to narrowly ovate, 10.0–15.0 × 4.0–6.0 cm	3–6 mm	4 or 5	12 or 13	Apex 3-lobed, apically cleft ca.2–3 mm, glabrous or sparsely pubescent	3-carpellate, densely tomentose	Puberulent, outer filaments basally connate for ca. 1/2 of their length
*C. debaoensis*	* Stereocarpus *	Oval to long oval, 6.0–13.0 × 2.5–5.0 cm	4(–6) mm	4 (or 5)	10	Apex 3-lobed, apically cleft to 1/6 style length, glabrous	3-carpellate, glabrous	Glabrous, outer filaments basally connate for ca. 1/4 of their length

**Table 2.** Morphological comparison of *C. debaoensis* with *C. pubipetala*, *C. mingii* and *C. tuyenquangensis*.

Items	*C. debaoensis*	*C. pubipetala*	*C. mingii*	*C. tuyenquangensis*
Young branches	Glabrous	Gray spreading villous	Densely spreading yellowish villous	Glabrous
Leaf	Ovate to long ovate, 6.0–13 × 2.5–5.0 cm, abaxial veins sparsely spreading villous, secondary veins 5–6	Elliptic-ovate, 10.0–17.0 × 5.0–8.0 cm, abaxial appressed villous but densely spreading villous along midvein, secondary veins 8–10	Elliptic-ovate to narrowly ovate, 10.0–15.0 × 4.0–6.0 cm, abaxial densely spreading villous along veins, secondary veins 7–10	Oblong ovate to narrow elliptic, 14.0–18.0 × 5.0–8.0 cm, abaxial glabrous, secondary veins 9–11
Petiole	5–12 mm long, glabrous	5–10 mm long, villous	5–7 mm long, densely villous	10–15 mm long, glabrous
Pedicel length	4 (–6) mm	3–5 mm	3–6 mm	10 mm
Sepals	Glabrous	Densely puberulent	Inside densely puberulent	Glabrous
Petals	10, glabrous	9–13, outside gray puberulent, inside glabrous	12 or 13, puberulent on both surfaces	12, glabrous
Stamens	2.0 cm long, glabrous	2.5–3.0 cm long, distinct part pilose	3.0 cm long, puberulent	2.5–3.0 cm long, glabrous
Ovary	Cylindrical, glabrous	Spherical, densely tomentose	Ovoid, densely tomentose	Ovoid, glabrous
Style	2 cm long, apex 3-lobed to 1/6 style length, glabrous	Apically 3 (or 4)-lobed to 1/3 style length, tomentose	3.0 cm long, apex 3-cleft to 1/10 style length, glabrous	3 cm long, apex 3-cleft to 1/2 style length, glabrous

### Key to identification of species of yellow camellia with connate style

**Table:** 

1	Ovary 3 carpellate, style 3-parted; if ovary 3 (or 4) carpellate, style 3 (or 4)-parted, young branches and leaves spreading villous (*C. pubipetala*)	**2**
–	Ovary 4–6 carpellate, style (3–) 4–6-parted	**8**
2	Petals 9–14	**3**
–	Petals 6	***C. capitata***
3	Young branches, petiole, petals, stamens, ovary and style piliferous	**4**
–	Young branches, petiole, petals, stamens, ovary and style glabrous	**5**
4	Petals puberulent on both sides, suborbicular	***C. mingii***
–	Petals adaxial puberulent, inside glabrous, elliptic	***C. pubipetala***
5	Petals with dense appressed brown hairs; style with dense white appressed hairs	***C. luteopallida***
–	Petals and style glabrous	**6**
6	Leaf 6–13 × 2.5–5 cm, abaxial veins sparsely spreading villous, secondary veins 5–6; ovary cylindrical; style apex 3-lobed to 1/6 the length of style	***C. debaoensis***
–	Leaf glabrous; style apex 3-cleft to 1/2 the length of style	**7**
7	Stamens 2.5–3 cm long; style 30 mm long	***C. tuyenquangensis***
–	Stamens 1.3–1.4 cm long; style 8–9 mm long	***C. thuongiana***
8	Leaf secondary veins 8–11; pedicel sessile, 1–5 mm long	**9**
–	Leaf secondary venation 24 pairs; pedicel 30–40 mm long	***C. oconoriana***
9	Petals 9–11, with margins sparsely ciliate; style finely tomentose	***C. bugiamapensis***
–	Petals 11–13, outer 5-petaloid concave; style glabrous	***C. luteocerata***

## Supplementary Material

XML Treatment for Camellia debaoensis
